# Modulation of Chloride Channel Functions by the Plant Lignan Compounds Kobusin and Eudesmin

**DOI:** 10.3389/fpls.2015.01041

**Published:** 2015-11-25

**Authors:** Yu Jiang, Bo Yu, Fang Fang, Huanhuan Cao, Tonghui Ma, Hong Yang

**Affiliations:** ^1^School of Life Sciences, Liaoning Provincial Key Laboratory of Biotechnology and Drug Discovery, Liaoning Normal UniversityDalian, China; ^2^College of Basic Medical Sciences, Dalian Medical UniversityDalian, China

**Keywords:** CFTR, CaCCs, ANO1/CaCC, kobusin, eudesmin, short-circuit current

## Abstract

Plant lignans are diphenolic compounds widely present in vegetables, fruits, and grains. These compounds have been demonstrated to have protective effect against cancer, hypertension and diabetes. In the present study, we showed that two lignan compounds, kobusin and eudesmin, isolated from *Magnoliae Flos*, could modulate intestinal chloride transport mediated by cystic fibrosis transmembrane conductance regulator (CFTR) and calcium-activated chloride channels (CaCCs). The compounds activated CFTR channel function in both FRT cells and in HT-29 cells. The modulating effects of kobusin and eudesmin on the activity of CaCC_gie_ (CaCC expressed in gastrointestinal epithelial cells) were also investigated, and the result showed that both compounds could stimulate CaCC_gie_-mediated short-circuit currents and the stimulation was synergistic with ATP. In *ex vivo* studies, both compounds activated CFTR and CaCC_gie_ chloride channel activities in mouse colonic epithelia. Remarkably, the compounds showed inhibitory effects toward ANO1/CaCC-mediated short-circuit currents in ANO1/CaCC-expressing FRT cells, with IC_50_ values of 100 μM for kobusin and 200 μM for eudesmin. In charcoal transit study, both compounds mildly reduced gastrointestinal motility in mice. Taken together, these results revealed a new kind of activity displayed by the lignan compounds, one that is concerned with the modulation of chloride channel function.

## Introduction

Plant lignans are widely distributed in vegetables, fruits, and grains, especially in rye, flax, and sesame seeds. It has been reported that lignans have various biological activities, including anti-cancer, anti-diabetic, antimicrobial, antiparasitic, and antihypertensive activities (Dar and Arumugam, 2013; Chun et al., 2014; Zhang et al., 2014a). However, little information is available on lignan compounds and chloride channels.

Active Cl^-^ secretion mediated by chloride channels provides a driving force for the transepithelial fluid secretion in the apical membrane of the intestines. It has been fully established that cystic fibrosis (CF) transmembrane conductance regulator (CFTR) and calcium-activated chloride channels (CaCCs) are the main chloride channels present in the luminal membrane of enterocytes (Riordan et al., 1989; Hartzell et al., 2005). CFTR is a cAMP-dependent chloride channel predominantly expressed in the crypt cells in the intestines, and is permeable to Cl^-^ and HCO_3_^-^ (Zhang et al., 2012). ANO1/CaCC (TMEM16A) is the first molecular identity of CaCCs that was found to express abundantly in the intestinal pacemaker Cajal cells, where it generates smooth muscle contraction (Huang et al., 2009; Hwang et al., 2009; Ferrera et al., 2010). CaCC_gie_ (CaCC that located in the gastrointestinal epithelial cells), which is CaCC apart from ANO1, is predominantly localized in the gastrointestinal epithelial cells and is involved in fluid secretion, though its molecular identity remains unclear.

Hyper activation of CFTR and CaCC proteins may account for such diseases as secretory diarrhea (Morris et al., 1999) and autosomal dominant polycystic kidney disease (Li et al., 2004), while dysfunction of these proteins may lead to CF (Kerem et al., 1989; Riordan et al., 1989), chronic pancreatitis (Cohn, 2005) as well as constipation (Morris et al., 1999). Chronic constipation (CC) is a common symptom characterized by infrequent stools and/or difficult stool passage (Lembo and Camilleri, 2003). The etiology of constipation is very complicated and may include diet, impaired colonic motility, behavioral and psychological factors (Lu et al., 2015). Current treatments are mainly based on dietary management and the use of laxatives, which usually show discouraging results. Therefore there is an urge to find new strategies for CC therapy. During the last decade, emphasis has been placed on increasing the intestinal fluid secretion and gastrointestinal motility as a new therapeutic option for the treatment of CC.

Previously, we have set up a high throughput screening strategy for identifying natural active compounds against chloride channels (Zhang et al., 2014b; Chen et al., 2015). Based on this strategy, we found a large number of compounds, including two lignan compounds, kobusin and eudesmin, which had CFTR and CaCC Cl^-^ channel modulation activities. The aim of the present study was to systematically investigate the modulation effects of kobusin and eudesmin on CaCCs and CFTR chloride channel activities. We demonstrated for the first time that plant lignan compounds could modulate intestinal chloride transport mediated by CFTR and CaCCs chloride channels.

## Materials and Methods

### Cell Lines, Animals, and Compounds

Cell lines used in this study were FRT (fischer rat thyroid epithelial) cells stably co-transfected with the YFP-H148Q fluorescence protein and human wild-type CFTR cDNA (Clarke et al., 2001; Harmon et al., 2010) or ANO1 cDNA (Hao et al., 2011) and HT-29 cells. FRT cells were cultured in Nutrient F12 coon’s medium (Sigma Chemical Co. St. Louis, MO, USA). HT-29 cells were cultured in 1640 medium (Sigma Chemical Co. St. Louis, MO, USA). Both media were supplemented with 10% fetal bovine serum (HyClone company, USA), 100 u/ml penicillin, 100 μg/ml streptomycin and 2 mM L-glutamine. The cells were incubated in a 5% CO_2_ incubator maintained at 37°C and 95% humidity before they were used for iodide influx fluorescence study and short-circuit current measurement.

Male ICR mice (8–10 weeks) were fed a standard chow diet and kept under specific pathogen-free conditions at Dalian Medical University (Permit Number: SCXK liao 2008-0002). All animal experiments were conducted in accordance with the Guide for the Care and Use of Laboratory Animals of the National Institutes of Health and were approved by the Liaoning Normal University Committee on Animal Research.

**FIGURE 1:**
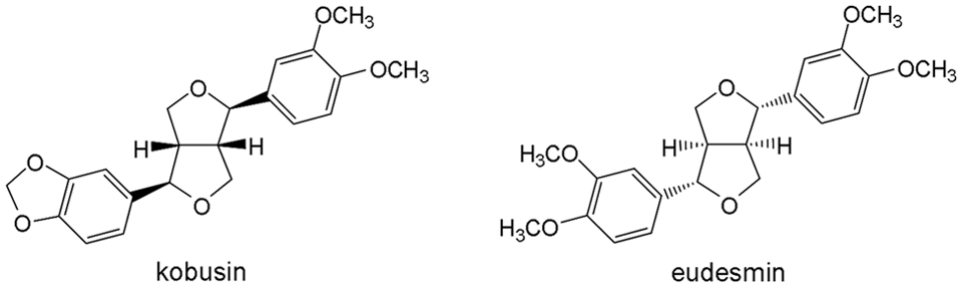
**Chemical structures of kobusin and eudesmin**.

CFTR_inh_-172 was synthesized as described previously (Garcia et al., 2009). Forskolin (FSK), genistein (Gen), indomethacin, amiloride and tannic acid were all purchased from Sigma (Sigma Chemical Co, St. Louis, MO, USA). Amphotericin B was purchased from Solarbio (Beijing Solarbio Science & Technology Co, Ltd). CaCC_inh_-A01 and E_act_ were obtained from Chembest Research Laboratory Limited (Shanghai). ATP and NaI were purchased from Sangon Biotech (Shanghai) Co, Ltd. Kobusin and eudesmin were isolated and purified in our own laboratory and their chemical structures are shown in **Figure 1**.

### Iodide Influx Fluorescent Assay

FRT cells transfected with CFTR were plated in a black 96-well plate with clear bottom (Costar, Corning, NY, USA) at a density of 2 × 10^4^ cells/well and incubated until confluent. The cells were washed three times with PBS followed by the addition of FSK (100 nM per well) and a further incubation of 5 min. After that, kobusin or eudesmin was added to each well at different concentrations and the cells were incubated for another 15 min. YFP fluorescence data were recorded using a FLUOstar Galaxy microplate reader (BMG Lab Technologies, Inc.) equipped with HQ500/20X (500 ± 10 nm) excitation, HQ 535/30M (535 ± 15 nm) emission filters (Chroma Technology Corp.) and syringe pumps. Iodide influx rates (d[I–]/dt) were computed as described by Kristidis et al. (1992).

### Short-circuit Current

Snapwell inserts containing ANO1-expressing FRT cells and HT-29 cells were mounted in Ussing chambers (Physiological Instruments, San Diego, CA, USA). For FRT cells, the hemi-chambers were filled with 5 ml of half-Cl^-^ solution (apical) and HCO_3_^-^ buffered solution (basolateral). The half-Cl^-^ solution contained 65 mM NaCl, 65 mM Na Gluconate, 2.7 mM KCl, 1.5 mM KH_2_PO_4_, 0.5 mM MgCl_2_, 2 mM CaCl_2_, 10 mM Hepes, 10 mM Glucose, and 25 mM NaHCO_3_ at pH 7.4. The HCO_3_^-^ buffered solution contained 120 mM NaCl, 5 mM KCl, 1 mM MgCl_2_, 1 mM CaCl_2_, 10 mM glucose, 5 mM Hepes, and 25 mM NaHCO_3_ at pH 7.4. Snapwell inserts were mounted in Ussing Chamber systems, with the resistance kept above 1500 Ω. Basolateral membrane was permeabilized with amphotericin B (250 μg/ml). For HT-29 cells, the Krebs’ buffered solution contained 130 mM NaCl, 2.7 KCl, 1.5 mM KH_2_PO_4_, 0.5 mM MgCl_2_, 2 mM CaCl_2_, 10 mM Hepes, 10 mM glucose at pH 7.4. Symmetrical HCO_3_^-^ buffered solutions contained 119 mM NaCl, 0.6 mM KH_2_PO_4_, 2.4 mM K_2_HPO_4_, 1.2 mM MgCl_2_, 1.2 mM CaCl_2_, 21 mM NaCO_3_, 10 mM glucose at pH 7.4. The cells were bathed in the buffered solution for 15 min at 37°C in the presence of 95% O_2_/5% CO_2_

Male ICR mice were sacrificed by an overdose of intraperitoneal sodium pentobarbital. The colon was removed as quickly as possible and washed with ice-cold modified Krebs-bicarbonate solution containing 120 mM NaCl, 5 mM KCl, 1 mM MgCl_2_, 1 mM CaCl_2_, 10 mM D-glucose, 5 mM Hepes, and 25 mM NaHCO_3_ at pH 7.4. After stripping of the muscularis, the tissue was mounted in an Ussing Chamber system (Physiological Instruments) connected to a VCC MC 6 multi-channel voltage-current clamp via silver/AgCl electrodes and 3 M KCl Ag bridges. The hemi-chambers were separately filled with 5 ml modified Krebs-bicarbonate solution bubbled with 95% O_2_/5% CO_2_ at 37°C. The hemi-chambers were filled with buffer solution containing 10 μM indomethacin to prevent the influence of prostaglandin, and the mucosal side of the tissue was exposed to 10 μM amiloride to inhibit epithelial Na^+^ current. Short-circuit current was recorded using Acquire and Analyze 2.3 software, with the transepithelial potential clamped at 0 mV during the whole experiment.

### Intestinal Motility Measurement

ICR mice were starved for 24 h and the animals were then orally administered PBS, 400 μM kobusin or eudesmin. Fifteen minutes later, the animals were administered 200 μl of 10% activated charcoal diluted in 5% gum Arabic. Thirty minutes after the administering of activated charcoal the animals were sacrificed and the small intestines were removed. Peristaltic index was calculated as the ratio of the length that activated charcoal traveled to the total length of the small intestine.

### Statistical Analysis

All data were expressed as mean ± SE or as representative traces. Student’s *t*-test was used to compare test and control values, and statistical significances were considered at the *P* < 0.05 level.

### Ethics Statement

This study was carried out in accordance with the recommendations of “Guide for the Care and Use of Laboratory Animals of the National Institutes of Health” and were approved by the Liaoning Normal University Committee on Animal Research. All surgery was performed under sodium pentobarbital anesthesia, and possible efforts were made to minimize suffering.

## Results

### Activation of CFTR Cl^-^ Channel Activity by Kobusin and Eudesmin

**FIGURE 2:**
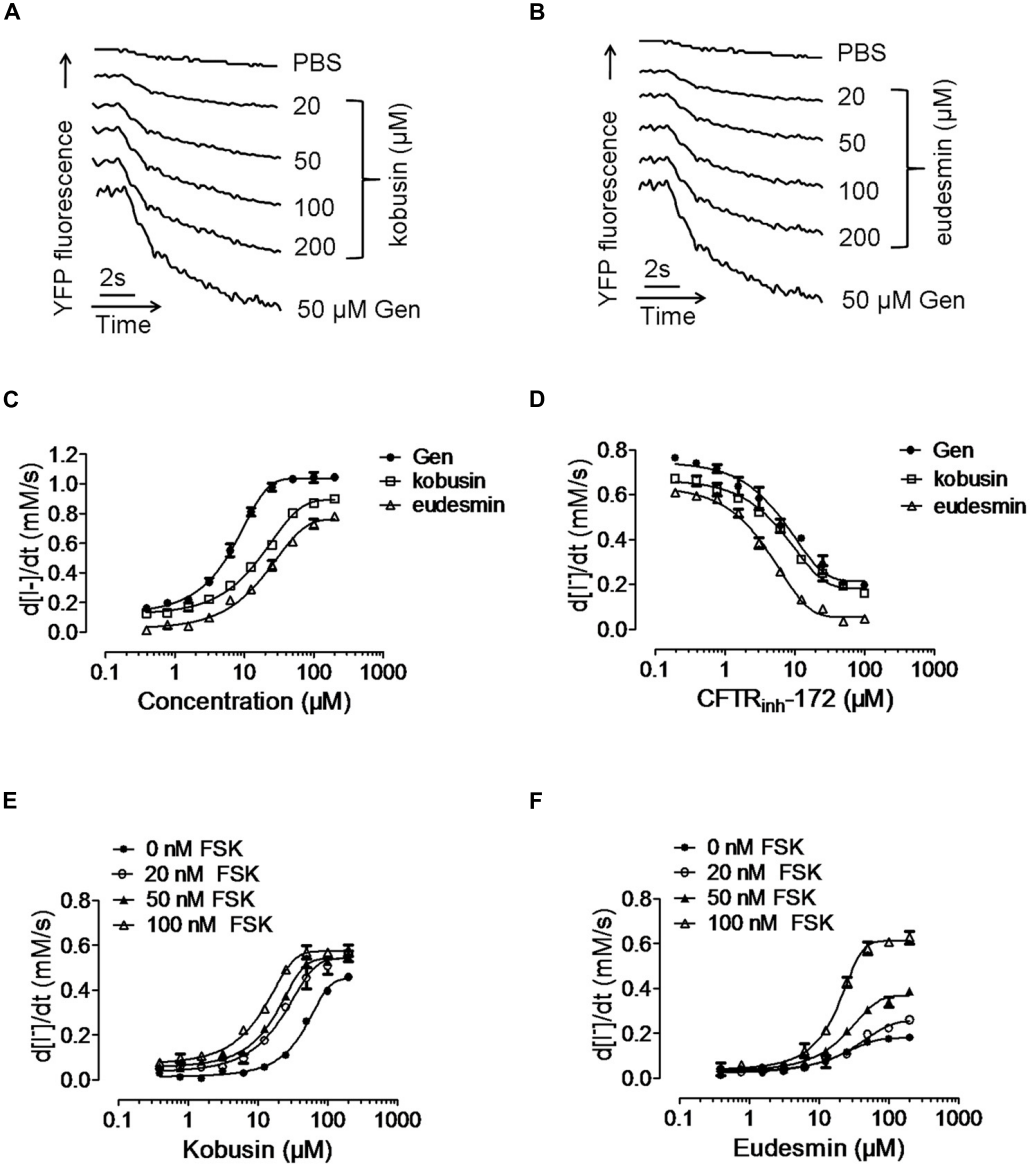
**Fluorescence quenching assay showing the activation of conductance regulator (CFTR) chloride channel activity by kobusin and eudesmin in transfected FRT cells.** **(A)** Original traces showing quenching of YFP fluorescence by I^-^ addition with PBS and after additions of indicated concentrations of kobusin and Gen (50 μM). **(B)** Original traces showing quenching of YFP fluorescence by I^-^ addition with PBS and after additions of indicated concentrations of eudesmin and Gen (50 μM). **(C)** Dose-dependent activation of CFTR chloride channel activity by kobusin and eudesmin. **(D)** Activation of CFTR Cl^-^ channel activity by kobusin and eudesmin in the presence of gradient concentrations of CFTR inhibitor CFTR_inh_-172. Dose-response effects of kobusin **(E)** and eudesmin **(F)** on the activation of CFTR in the presence of different concentrations of FSK. Data are the means ± SEs of three independent tests.

Activation effect of kobusin or eudesmin on CFTR chloride channel activities were tested using a cell-based fluorescence assay using FRT cells transfected with human CFTR cDNA (Ma et al., 2002). A known CFTR activator Gen (Hwang et al., 1997) was used as a positive control. FSK (100 nM) was added to the cells to acquire a basal level of cAMP (**Figures 2A,B**). Kobusin and eudesmin activated CFTR chloride channel activity in a dose-dependent manner with EC_50_ values of 30 and 50 μM, respectively, for kobusin and eudesmin (**Figure 2C**). Further experiments showed that the activation effect of these compounds could be inhibited by gradient concentrations of the known CFTR inhibitor CFTR_inh_-172 (**Figure 2D**). CFTR activation can be achieved by direct interaction with CFTR protein or activation of upstream cAMP-dependent PKA signaling pathway (Hwang and Sheppard, 1999; Schultz et al., 1999; Sheppard and Welsh, 1999). To investigate the mechanisms involved in the activation, we measured the activities of kobusin and eudesmin under different FSK concentrations. Kobusin was effective at inducing CFTR-mediated iodide influx in the absence of FSK, although the potency was relatively weaker than that in the presence of FSK (**Figure 2E**). On the other hand, activation of CFTR by eudesmin depended on cAMP level and phosphorylation level of CFTR more than kobusin, which is that eudesmin showed a stronger activation effect under high concentrations of FSK (**Figure 2F**). The results suggested that eudesmin’s efficacy is more dependent on the phosphorylation level of CFTR than kobusin.

### Activation of CFTR Chloride Channel Activities by Kobusin and Eudesmin in HT-29 Cells

**FIGURE 3:**
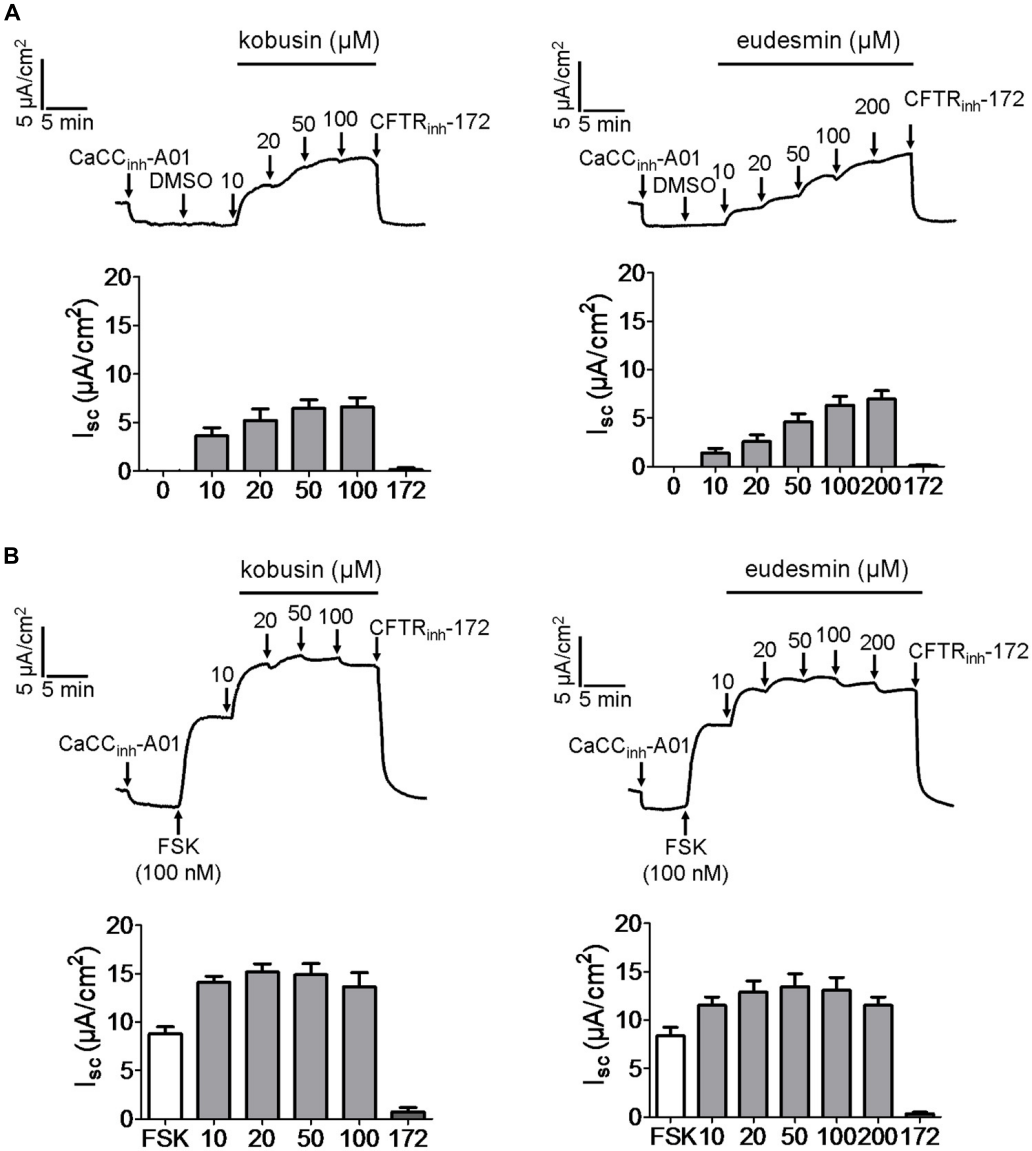
**Activation of CFTR chloride channel activities by kobusin and eudesmin in HT-29 cells.** **(A)** Activation effect of CFTR chloride channel activity by kobusin and eudesmin without FSK. **(B)** Activation of CFTR chloride channel activity by kobusin and eudesmin in the presence of 100 nM FSK. CaCC_inh_-A01 (30 μM), DMSO (100 nM) and CFTR_inh_-172 (50 μM) were added where indicated. Histograms showing summary of short-circuit currents induced by kobusin and eudesmin. 172: CFTR_inh_-172. Data are the means ± SEs of three independent tests.

CFTR and CaCCs are endogenously expressed in HT-29 cells (Morris and Frizzell, 1993), and therefore, short-circuit current experiment was performed to investigate the kobusin- and eudesmin-induced activation effect on CFTR chloride channel activity in HT-29 cells. All tests were done in the presence of 30 μM of the CaCC-specific inhibitor CaCC_inh_-A01 (De La Fuente et al., 2008) to eliminate the influence of endogenous CaCC current. **Figure 3A** shows that both kobusin and eudesmin alone could increase the CFTR-mediated short-circuit currents in a dose-dependent manner. Kobusin and eudesmin both elicited more potent CFTR-mediated short-circuit currents in the presence of 100 nM FSK (**Figure 3B**). Summarized data are shown in **Figures 3A,B** (down panels).

### Potentiation of CaCC Chloride Channel Activity by Kobusin and Eudesmin in HT-29 Cells

**FIGURE 4:**
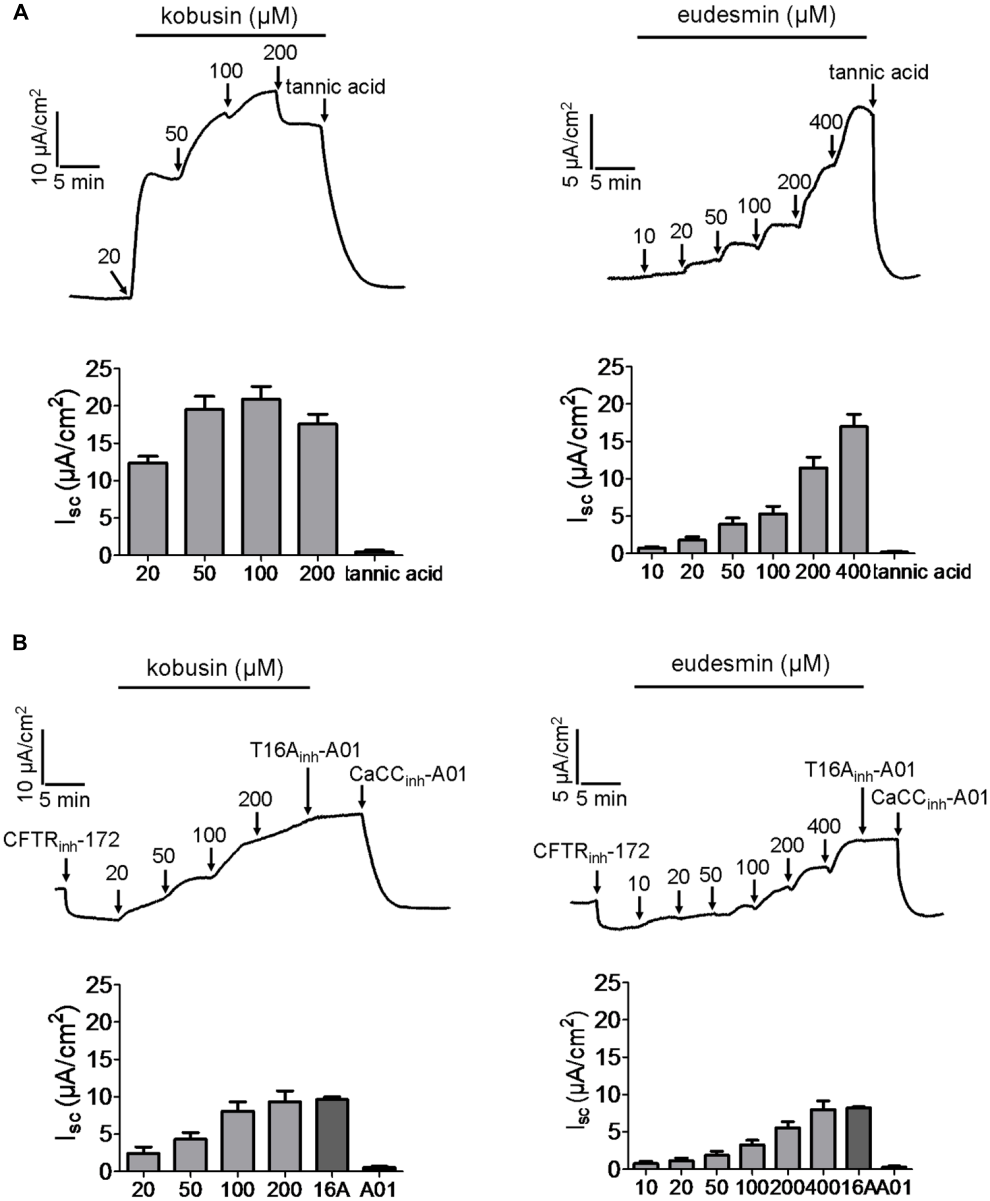
**Activation of Ca^2+^-activated chloride channel activities by kobusin and eudesmin in HT-29 cells expressing endogenous CaCC_gie_.** **(A)** Representative current traces showing kobusin (left panel) and eudesmin (right panel) -stimulated Cl^-^ current. The activation effect was abolished by tannic acid (100 μM). **(B)** Representative traces showing kobusin (left panel) and eudesmin (right panel) -induced CaCC-mediated Cl^-^ current. The activation effect was abolished by CaCC_inh_-A01 (30 μM). Histograms showing summary of kobusin and eudesmin-induced short-circuit currents. 16A: T16A_inh_-A01. A01: CaCC_inh_-A01. Data are the means ± SEs of three independent tests.

As CaCC_gie_ is endogenously expressed in HT-29 cells (Morris and Frizzell, 1993), we wanted to know what effect kobusin and eudesmin would exert on this kind of chloride channel. **Figure 4A** indicates that both kobusin and eudesmin could activate short-circuit current, and the activation effect could be abolished by the known non-specific CFTR and CaCC inhibitor tannic acid (100 μM). After pretreatment with CFTR_inh_-172 (20 μM), kobusin and eudesmin further activated the short-circuit current, and the activation effect was inhibited by the specific CaCC inhibitor CaCC_inh_-A01 (**Figure 4B**), suggesting that both compounds were able to activate CaCC-mediated Cl^-^ current.

**FIGURE 5:**
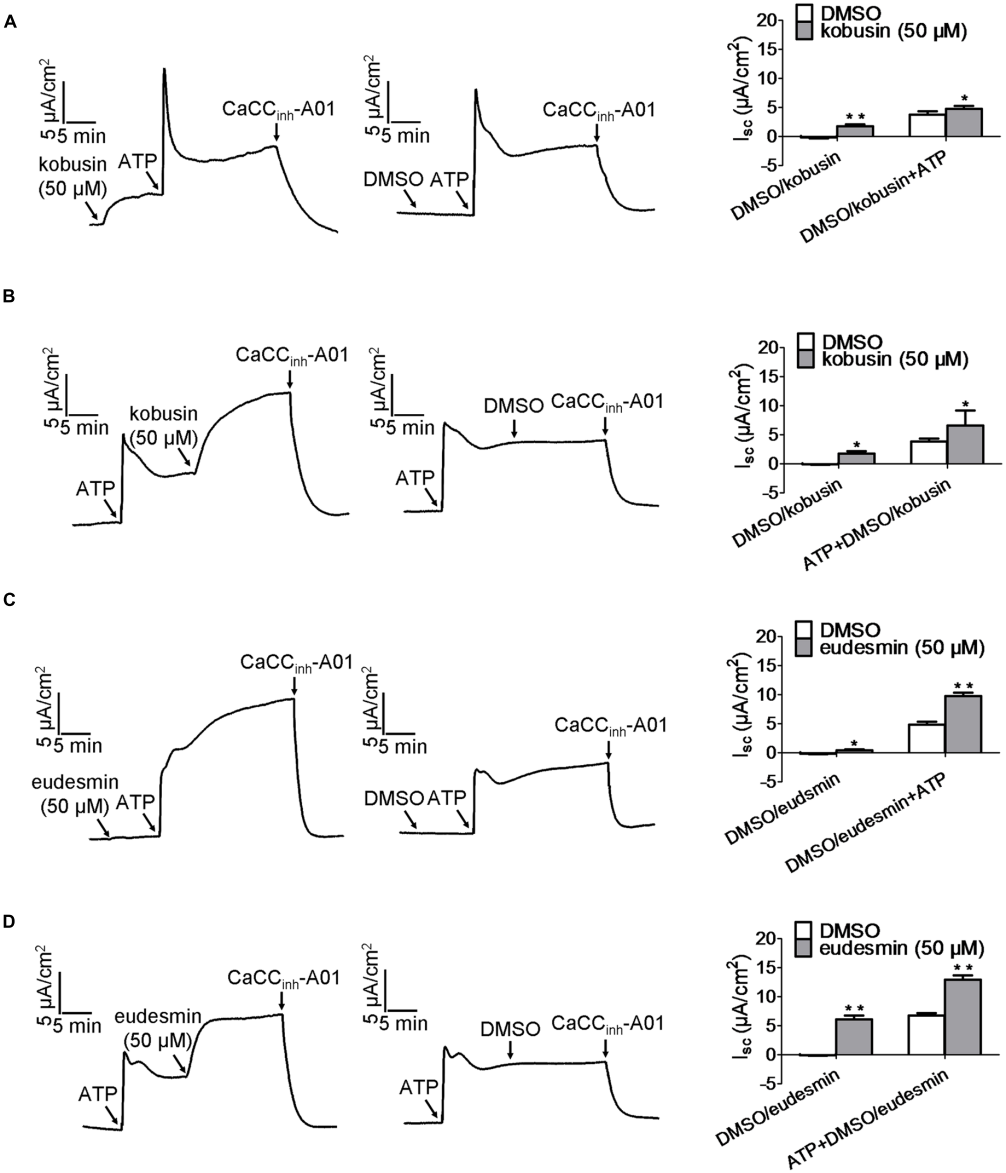
**Characteristic of CaCC_gie_ potentiation by kobusin and eudesmin in HT-29 cells.** **(A,B)** Representative traces of short-circuit currents showing synergistic effect between ATP and kobusin on the potentiation of CaCC_gie_ chloride channel. ATP was added after **(A)** and before **(B)** incubation with 50 μM kobusin. **(C,D)** Representative traces of short-circuit currents showing synergistic effect between ATP and eudesmin on CaCC_gie_ chloride channel potentiation. ATP was added after **(C)** and before **(D)** incubation with the indicated 50 μM eudesmin. CaCC_gie_ currents were abolished with CaCC_inh_-A01 at the end of tests. Statistical data are shown in the right panel of **(A–D)**. Data are the means ± SEs of three independent tests. ^∗^*P* < 0.05, ^∗∗^*P* < 0.01.

To evaluate whether the two lignan compounds activated CaCC_gie_ the way ATP does, we measured the activation effects of the lignan compounds added before and after the addition of ATP. Kobusin and eudesmin induced a higher short-circuit current than DMSO (which served as a control) regardless of whether the compound was added before or after the addition of ATP (**Figure 5**). Kobusin (50 μM) induced a higher short-circuit current than 50 μM DMSO (which served as a control) and achieved a short-circuit current increase of 2.6-folds at 50 μM when kobusin was added before addition of ATP (**Figure 5A**). When added after addition of ATP, kobusin achieved a short-circuit current increase of 3.7-folds at 50 μM (**Figure 5B**). Similar to kobusin, eudesmin achieved a short-circuit current increase of 2.4-folds and at 50 μM when added before addition of ATP (**Figure 5C**). When added after addition of ATP, eudesmin achieved a short-circuit current increase of 2.15-folds at 50 μM (**Figure 5D**). The results thus indicated the presence of synergistic effect between kobusin or eudesmin and ATP.

### Inhibitory Effects of ANO1 Chloride Channel Activities by Kobusin and Eudesmin in ANO1-expressing FRT Cells

**FIGURE 6:**
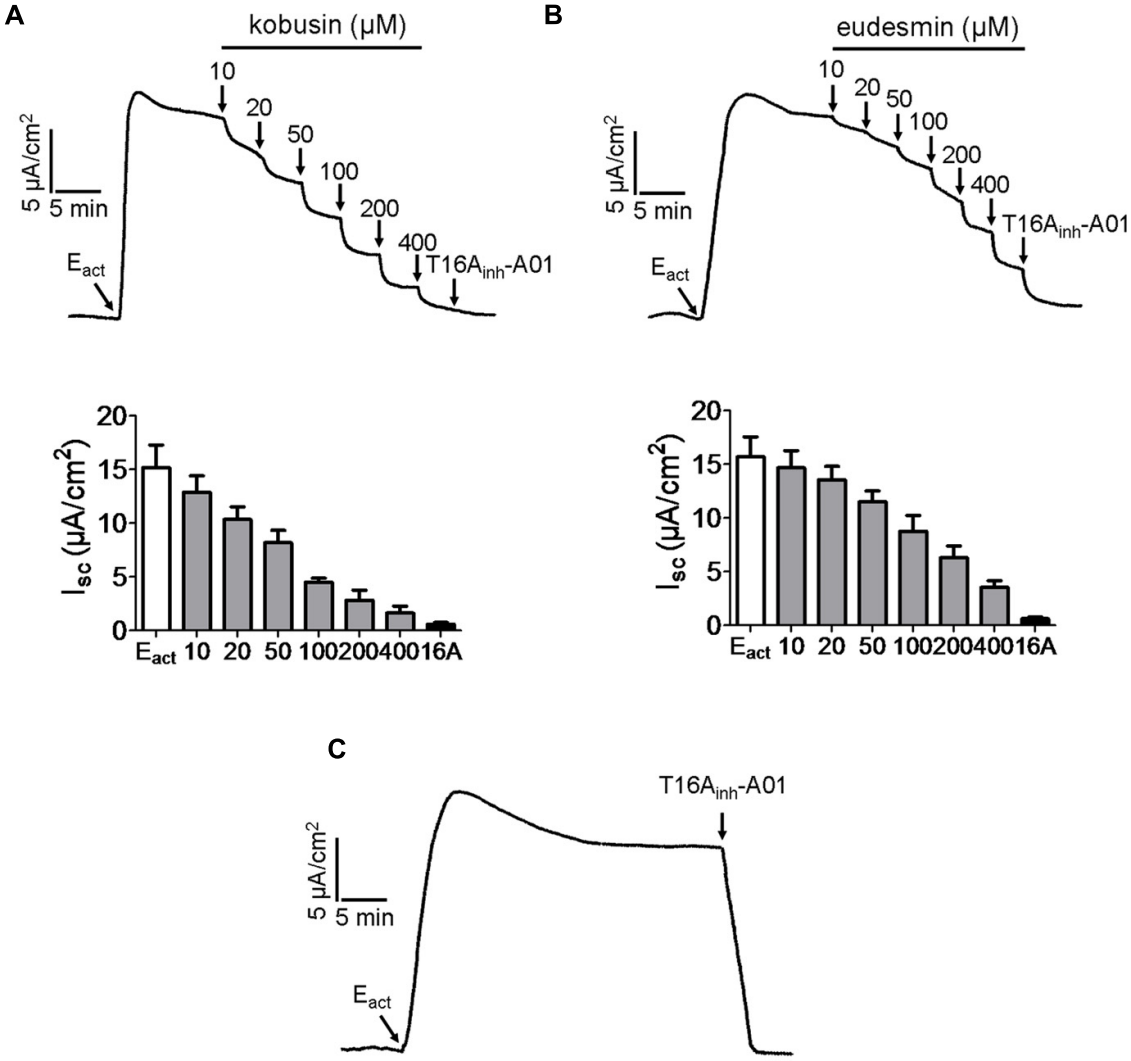
**Inhibitory effects of kobusin and eudesmin on ANO1-mediated short-circuit currents in ANO1-expressing FRT cells.** **(A)** Dose-dependent inhibition of E_act_-induced ANO1-mediated short-circuit current by kobusin. Summary data are shown in down panel. **(B)** Inhibition of ANO1 chloride channel activity by different concentrations of eudesmin. Summary data are shown in down panel. **(C)** Inhibition of ANO1 chloride channel activity by T16A_inh_-A01 (30 μM). Apical membrane current was measured in the presence of a transepithelial Cl^-^ gradient and after basolateral membrane permeabilization. Data are the means ± SEs of three independent tests. 16A: T16A_inh_-A01.

ANO1 is the first identified molecular component of CaCCs, and thus we investigated the effects of kobusin and eudesmin on ANO1 chloride channel activity. E_act_ Namkung et al. (2011b) was used to produce an ANO1-mediated short-circuit current, followed by indicated concentrations of kobusin or eudesmin additions. The remaining currents were abolished by T16A_inh_-A01 (Namkung et al., 2011a). The results showed that apical application of kobusin and eudesmin inhibited E_act_-induced ANO1-mediated short-circuit currents in transfected FRT cells in a dose-dependent manner with IC_50_ values of 100 μM for kobusin (**Figure 6A**) and 200 μM for eudesmin (**Figure 6B**). Statistical analysis is shown in **Figures 6A,B** (down panels). E_act_-induced ANO1-mediated short-circuit current was completely abolished by the specific inhibitor of ANO1 T16A_inh_-A01 (**Figure 6C**).

### Activation of CFTR and CaCC Chloride Channel Activities by Kobusin and Eudesmin in Mouse Colonic Epithelia

**FIGURE 7:**
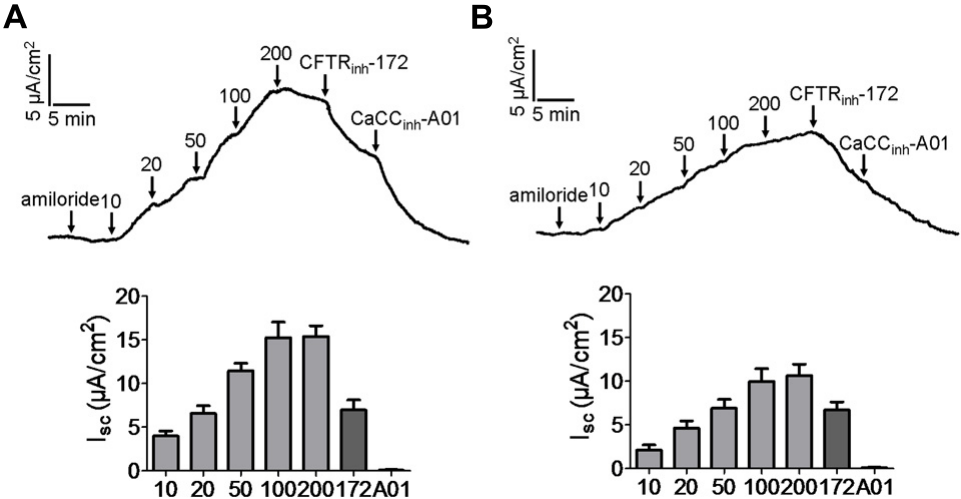
**Activation of CFTR and CaCC-mediated short-circuit Cl^-^ current by kobusin and eudesmin in mouse colonic mucosa.** **(A)** Activation of CFTR and CaCC by kobusin. Kobusin was added to the mucosal side of the tissue at the indicated concentrations. **(B)** Activation of CFTR and CaCCs by eudesmin. Eudesmin was added to the mucosal side of the tissue at the indicated concentrations. Addition of CFTR_inh_-172 (100 μM) and CaCC_inh_-A01 (100 μM) in the end of the experiments were added to completely inhibit the activation effect of the lignans. The traces shown are typical result of three independent experiments. Histograms showing summary data of short-circuit currents induced by kobusin and eudesmin. 172: CFTR_inh_-172. A01: CaCC_inh_-A01. Data are the means ± SEs of three independent tests.

As CFTR and CaCCs are the major pathway for apical Cl^-^ exit in the intestine, the efficacies of kobusin and eudesmin were tested *ex vivo* in isolated mouse colonic mucosa by short-circuit current analysis. The experiments were performed in the presence of 10 μM indomethacin and 10 μM amiloride to eliminate the influence of prostaglandin generation and Na^+^ transport. Kobusin and eudesmin increased the short-circuit currents in a dose-dependent manner in mouse colonic epithelia (**Figures 7A,B**). As expected, the activation effect was completely abolished by 100 μM CFTR_inh_-172 plus 100 μM CaCC_inh_-A01, but was only partially inhibited by CFTR_inh_-172 or CaCC_inh_-A01 alone.

### Inhibition of Intestinal Motility by Kobusin and Eudesmin

**FIGURE 8:**
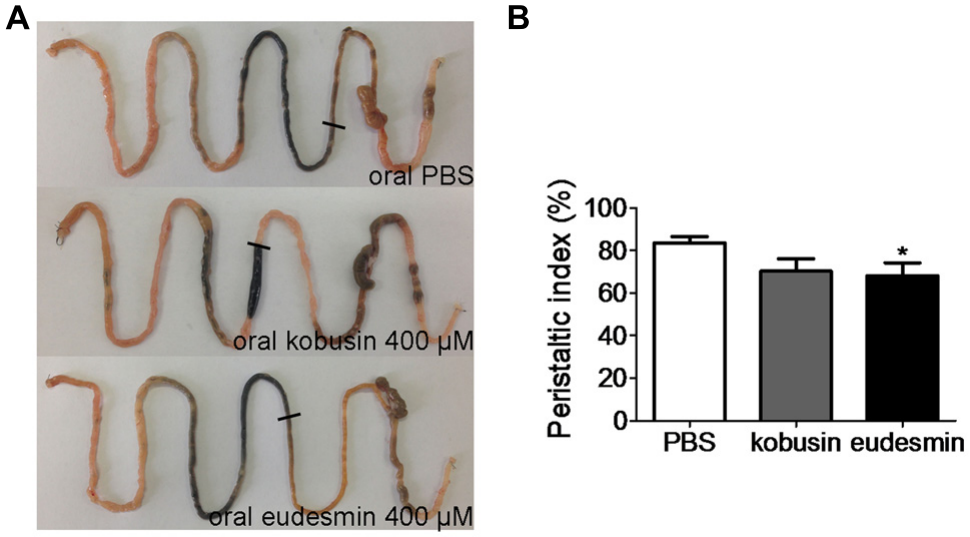
**Inhibition of gastrointestinal motility by kobusin and eudesmin in mice.** **(A)** Photographs of isolated mouse intestinal tracts showing the distance traveled by activated charcoal after oral gavage of PBS (negative control) or kobusin and eudesmin. **(B)** Summary of the peristaltic indexes. Data are the means ± SEs of three independent experiments. ^∗^*P* < 0.05.

Since ANO1 is expressed in the pacemaker cells that generate smooth muscle contraction in the gastrointestinal tract (Huang et al., 2009; Hwang et al., 2009; Ferrera et al., 2010), more experiments were performed *in vivo* to evaluate the inhibitory effects of kobusin and eudesmin on gastrointestinal motility. Oral administration of either kobusin or eudesmin inhibited intestinal peristalsis and delayed charcoal movement in mice, with peristaltic indexes of 70.6 ± 5.9% in the case of kobusin and 68.2 ± 5.9% in the case of eudesmin, compared to that of PBS (82.3 ± 2.9%; **Figures 8A,B**).

## Discussion

Lignans, described as a group of diphenolic compounds where the C6-C3 carbons are bound by the C8 central carbon, are widely distributed in more than 70 families of vascular plants. They have been isolated from different parts of a plant, including roots, stems, rhizomes, leaves, seeds, and fruits as well as the exudates and resins (Gang et al., 1997; Pan et al., 2009). Although neither non-nutrient nor non-caloric, lignans have attracted considerable attention because of their various biological activities. Numerous studies have shown that lignans and their intestinal metabolites enterolignans possess antitumor, antiviral, and antioxidant activities (Chen et al., 1997; Ashakumary et al., 1999; Saarinen et al., 2002). Furthermore, they have also been shown to have osteoporosis prevention and liver-protection activities as well as antagonistic activity toward platelet-activating factor (PAF; Han et al., 1992; Habauzit and Horcajada, 2008). In the present study, we demonstrated that two lignan compounds, kobusin and eudesmin, could function as activators of CFTR and CaCC_gie_ chloride channels and inhibitors of ANO1/CaCC channel. The results revealed that kobusin and eudesmin could activate the function of CFTR and CaCC_gie_ chloride channels. Notably, we found that kobusin and eudesmin could inhibit the activities ANO1/CaCC chloride channel in ANO1/CaCC-expressing FRT cells, and reduce gastrointestinal motility in mice, thereby uncovering new molecular pharmacological targets of lignan compounds.

The identification of lignan compounds as CFTR and CaCC_gie_ activators would highlight their potential uses as lead drugs for the treatment of constipation. Intestinal fluid secretion provides a proper environment for digestion and facilitates stool passage through the intestinal tract (Barrett and Keely, 2000), and this process is driven by chloride channel-mediated Cl^-^ transport in the enterocyte (Barrett and Keely, 2000; Kiela and Ghishan, 2009). So far, three chloride channels (namely CFTR, CaCC, and ClC-2) have been identified to mediate Cl^-^ secretion into the intestinal lumen side, and among these, CFTR and CaCC play pivotal roles (Murek et al., 2010). In the intestine, CFTR, a cAMP-activated chloride channel, is mainly expressed in the crypt (Zhang et al., 2012). Mutation in the CFTR protein (e.g., ΔF508-CFTR) may result in the hereditary lethal disease of CF (Lubamba et al., 2012). Habitual constipation remains a common symptom among CF patients, which is regarded as a consequence of impaired intestinal fluid secretion (Grubb and Gabriel, 1997). Since the cloning of the CFTR gene back in 1989 (Riordan et al., 1989), CFTR has been advocated as a potential therapeutic molecular target for the treatment of several diseases, including CF (Kerem et al., 1989; Riordan et al., 1989), chronic pancreatitis (Cohn, 2005), habitual constipation (Morris et al., 1999), secretory diarrhea and autosomal dominant polycystic kidney disease (Li et al., 2004). Though the molecular identity of CaCC_gie_ still remains elusive, its existence in enterocyte has been fully confirmed (De La Fuente et al., 2008). CaCC_gie_ is responsible for rotaviral enterotoxin-stimulated diarrhea (Ko et al., 2014). In the present study, we demonstrated that kobusin and eudesmin could potentiate the two major pathways of Cl^-^ secretion, suggesting that mild activation of CFTR and CaCC_gie_ may result in a significant activation of fluid secretion in the intestine. However, kobusin and eudesmin also inhibited the activity of ANO1/CaCC chloride channel (**Figure 6**). Kobusin and eudesmin inhibited ANO1/CaCC-mediated short-circuit current in transfected FRT cells, and reduced gastrointestinal motility in mice. ANO1/CaCC is highly expressed in the pacemaker Cajal cells of the gastrointestinal tract (Huang et al., 2009; Hwang et al., 2009; Ferrera et al., 2010). Inhibition of ANO1/CaCC may delay the movement of the intestine and thus increase the fluid absorption time (Hwang et al., 2009). Thus the neutralization effect of these compounds on fluid secretion needs to be fully considered.

The study of lignan supplementation in some randomized controlled trials has indicated that lignans cause a mild but significant reduction in diastolic or/and systolic blood pressure in patients with hypertension (Ursoniu et al., 2015). Raimundo et al. (2009) reported that eudesmin can induce endothelium-dependent relaxation in rat aorta. The molecular mechanism of this effect still remains elusive. Accumulating evidence suggests that ANO1/CaCC plays important roles in the pathogenesis of spontaneous hypertension. Moreover, inhibition of ANO1/CaCC channel activity can reduce blood pressure in rodents so that spontaneous hypertension can be inhibited (Wang et al., 2015). The fact that kobusin and eudesmin could inhibit the activity of ANO1/CaCC channel both *in vitro* and *in vivo* suggested that inhibition of ANO1/CaCC may in part account for the antihypertension activities of the lignans.

Both epidemiological data and experimental evidence have indicated that lignans or lignan-rich food possess anti-carcinogenic activities against many types of cancer, including breast (Saarinen et al., 2007), prostate (McCann et al., 2005), and colon (Webb and McCullough, 2005) cancers. The mechanisms involved in the cancer prevention effects are related to the anti-estrogenic, anti-angiogenic, pro-apoptotic, and anti-oxidant activities of these compounds (Webb and McCullough, 2005). Recently, it has been confirmed that ANO1/CaCC is overexpressed in several tumors (Qu et al., 2014; Wanitchakool et al., 2014), and inhibition of ANO1/CaCC channel activity may suppress the proliferation and migration of cancer cells (Sui et al., 2014; Seo et al., 2015). The inhibition effect of kobusin and eudesmin on ANO1/CaCC channel activities observed in this study may provide new insight into the molecular mechanism associated with the anticancer effect of lignans.

The botanical properties of lignans have not been unveiled. Although prevalent in plants, lignans are virtually not found in animals (Peterson et al., 2010). The biosynthesis pathways of lignans are thought to have evolved in plants during their adaptation to the land (Davin and Lewis, 2000). Accumulating evidence shows that lignans are produced by phenoxy-radical coupling and polymerization, in which the dirigent proteins play key roles in determining the regiospecificity and stereoselectivity of the compounds (Gang et al., 1999), while the precise molecular mechanism is still unclear. In general, the lignan content in food is low except for flax seed, rye bran, and sesame seeds (Hallmans et al., 2003; Peterson et al., 2010). The demand for lignans has been increasing rapidly. The inefficiency and instability of plant lignan production means that there is an urgent need for a new technology to produce lignan. Recent studies have shed light on the production of lignans using transgenic plants and cells (Satake et al., 2013). Previous study has reported that ATP can activate CaCC chloride channel activities through both PLC and intracellular Ca^2+^ pathways (Rajagopal et al., 2011). We detected a synergistic effect between kobusin or eudesmin and ATP, which suggested that these compounds activated CaCC_gie_ Cl^-^ channel function in a way that may differ from ATP. Furthermore, we would like to know how these two lignan compounds inhibited ANO1 chloride channel function. Since CaCCs are Ca^2+^-activated chloride channels, the inhibition of Ca^2+^ release may impair the function of ANO1/CaCC or CaCC_gie_ channels. Zhang et al. (2013) reported that the lignan compound magnolol can inhibit colonic motility through down-regulating the voltage-sensitive L-type Ca^2+^ channel activities in rat colonic smooth muscle cells. Although both FRT and HT-29 cells express L-type calcium channels (Montiel et al., 2001; Perego et al., 2012), the adverse effect of kobusin and eudesmin on CaCC_gie_ and ANO1 chloride channel activities observed in our study did not support the L-type Ca^2+^ channel inhibition pathway. The detailed mechanisms will need further investigation.

## Conclusion

The present study discovered the modulation of chloride channel function as a new activity of the lignan compounds kobusin and eudesmin, thereby uncovering new insights into the mechanism relating to the antihypertension and cancer prevention activities of lignans in general.

## Conflict of Interest Statement

The authors declare that the research was conducted in the absence of any commercial or financial relationships that could be construed as a potential conflict of interest.
